# Past, present and future contributions of evolutionary biology to wildlife forensics, management and conservation

**DOI:** 10.1111/eva.12977

**Published:** 2020-05-13

**Authors:** Vincent Bourret, Vicky Albert, Julien April, Guillaume Côté, Olivier Morissette

**Affiliations:** ^1^ Direction générale de la protection de la faune Ministère des Forêts, de la Faune et des Parcs Québec QC Canada; ^2^ Direction générale de la gestion de la faune et des habitats Ministère des Forêts, de la Faune et des Parcs Québec QC Canada

**Keywords:** conservation, eDNA, forensic, genetics, genomics, management, wildlife

## Abstract

Successfully implementing fundamental concepts into concrete applications is challenging in any given field. It requires communication, collaboration and shared will between researchers and practitioners. We argue that evolutionary biology, through research work linked to conservation, management and forensics, had a significant impact on wildlife agencies and department practices, where new frameworks and applications have been implemented over the last decades. The Quebec government's Wildlife Department (MFFP: *Ministère des Forêts, de la Faune et des Parcs*) has been proactive in reducing the “research–implementation” gap, thanks to prolific collaborations with many academic researchers. Among these associations, our department's outstanding partnership with Dr. Louis Bernatchez yielded significant contributions to harvest management, stocking programmes, definition of conservation units, recovery of threatened species, management of invasive species and forensic applications. We discuss key evolutionary biology concepts and resulting concrete examples of their successful implementation that derives directly or indirectly from this successful partnership. While old and new threats to wildlife are bringing new challenges, we expect recent developments in eDNA and genomics to provide innovative solutions as long as the research–implementation bridge remains open.

## 1 INTRODUCTION

**FIGURE 1:**
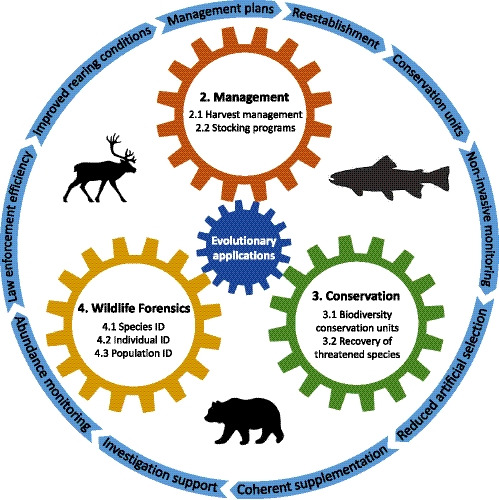
Schematic of the article's themes (sections and subsections) showing the implementation outcomes set in motion by the integration of evolutionary applications in MFFP practices

Over the last decade, there have been recurrent discussions over the existence of a more or less wide “research–implementation gap” for evolutionary biology in the wildlife sciences (Cook & Sgro, 2019; Knight et al., 2008). While it is relatively well accepted that traditional genetic applications have made their way into practitioners’ tool bags, whether narrow or broad sense genomics concepts have leapt successfully over the gap remains debated (Garner et al., 2016; Shafer et al., 2015). Nonetheless, a consensus appears to exist around the idea that communication, collaboration and integration of evolutionary biology in students’ undergraduate studies are key to linking the work of academics and wildlife practitioners (Cash et al., 2003; Hogg et al., 2017; Shafer et al., 2015). Quebec's (Canada) Wildlife Department (MFFP: *Ministère des Forêts, de la Faune et des Parcs*), where the authors are currently employed, has benefited from a strong association with academics to fulfil its mission, particularly through an extensive collaboration with Dr. Louis Bernatchez's laboratory at Laval University (Box 1). In regard to wildlife, the mission of our department translates into concerted efforts towards sustainable wildlife and habitat conservation using the best available practices. Although this may be true for a variety of fields, such as telemetry, climatology and geology to name a few, we argue that over the last decades, none has experienced faster growth or a wider impact than genetics and more recently, genomics in addressing otherwise tedious and integrative questions in conservation biology. Hence, the MFFP greatly benefits from the maturity of genetics and genomics in three important fields of expertise, namely wildlife management, conservation and forensics (Figure 1).

Box 1 **Progressive implementation of evolutionary applications in wildlife management in Quebec—Michel Legault (fishery biologist, MFFP)**

The application of evolutionary and genetic concepts in aquatic wildlife management before the 1990s was limited to using species nomenclature in the conservation of threatened species and the regulation of angling and hunting. Only after the democratization of genetic methods and the increased accessibility to microsatellite and AFLP (amplified fragment length polymorphism) data for the analysis of population structure did the integration of evolutionary applications in wildlife management expand. This new expertise encouraged the emergence of wildlife genetic specialists working in both fundamental and applied research. This period was also the beginning of multiple collaborations between academic geneticists (Julian Dodson and Louis Bernatchez, to name only a couple) and MFFP wildlife managers to exploit this new potential for acquiring knowledge that could refine management decisions. Such collaborations were critical in facilitating (with all the challenged it represented) the understanding, recognition and implementation of this field in our department.

Today, biologists working in wildlife management have been trained in an “evolutionary‐enlightened” context and have access to multiple molecular tools to meet their needs. Recent works, exemplified here by many cited article, are good examples of evolutionary applications that provided data to guide the development of revised management practices, new conservation strategies and more efficient law enforcement to name a few. Such projects would not have been possible without past efforts in education and implementations by governmental and academic teams. Lately, the constant increase in new molecular tools implementation (i.e., environmental DNA) in our department makes it clear that evolutionary applications will continue to be a valuable contribution and represent an important aspect of wildlife management.

From conceptual research and technical developments, many applications have emerged into specific fields of applied evolutionary biology. The genetic structure of populations has been described for a plethora of systems worldwide, allowing for a more precise definition of management units that is based on connectivity, genetic diversity and effective size (Palsboll, Berube, & Allendorf, 2007; Waples & Naish, 2009; Yannic et al., 2016). Moreover, knowledge of genetic differentiation among groups of exploited populations can be used to estimate their respective contributions to exploitation (Utter & Ryman, 1993). Genetic diversity metrics are also being used to identify source and sink populations to better plan local supplementations, genetic rescue actions or reintroductions (Whiteley, Fitzpatrick, Funk, & Tallmon, 2015). Practitioners now recognize that to have a successful recovery of a population, molecular analyses should be integrated to define the necessary level of resources to be committed (Haig et al., 2016). Molecular ecology can also serve as an assessment tool for population‐specific management plans; for example, it can evaluate the contribution of a captive breeding programme to population recovery (Thériault, Moyer, Jackson, Blouin, & Banks, 2011) or effects of artificial selection induced by restricted size exploitation (Hutchings, 2009).

Undoubtedly, biodiversity conservation is fundamental and depends on the recognition of taxonomic diversity as well as the threats to habitats and vulnerable species. From phenotypic observations, taxonomy has been complemented, although admittedly sometimes challenged, by worldwide initiatives of genetic identification confined in barcode reference databases (e.g., The Barcode of Life Data System; www.boldsystems.org, Ratnasingham & Hebert, 2007). Such initiatives have identified cryptic species and systems of unsuspected evolutionary potential (April, Mayden, Hanner, & Bernatchez, 2011; Janzen et al., 2017). While some advocate for a more comprehensive approach for maintaining the evolutionary potential of biodiversity (Milot et al., this issue), including this evolutionary potential in a practitioner's baseline criteria is a tangible manifestation of the recent effect of evolutionary biology in conservation science. Alternatively, conservation can rely on nonrecombinant genetic markers to investigate historical, rather than contemporary, forces that have shaped the foundations of present‐day diversity and further outline the uniqueness of the recovery path for a particular species (Bernatchez, 1997).

Wildlife forensic sciences have also benefited from the expansion of evolutionary applications. To support investigations of illegal activities related to wildlife, such as poaching as well as local and international trade, wildlife officers rely on scientific expertise from various fields that include ballistics, veterinary sciences, morphology, chemistry and, most importantly, molecular applications. As wildlife forensics deals with a variety of species‐specific laws and regulations based on local, regional, national and international legislation, species identification has become a routine analysis (Linacre & Ciavaglia, 2017). While morphological analyses are usually low cost and have a fast turnover if an expert of the taxonomic group of interest is at hand, genetic identification methods such as DNA barcoding are more convenient when dealing with ambiguous or incomplete specimens (Ogden, 2010). Human forensics have inspired many wildlife forensic applications, and there is an expanding set of genetic markers developed for and applied to individual identification purposes (e.g., Andreassen et al., 2012; Ciavaglia & Linacre, 2018). Such expertise allows a random match probability to be calculated based on estimated allele frequencies and the population structure for a given species. Furthermore, as the liberalization of molecular biology and genomic methods progresses, we expect to see more frameworks developed for identifying an individual's population or geographic origin (Ogden & Linacre, 2015).

**FIGURE 2:**
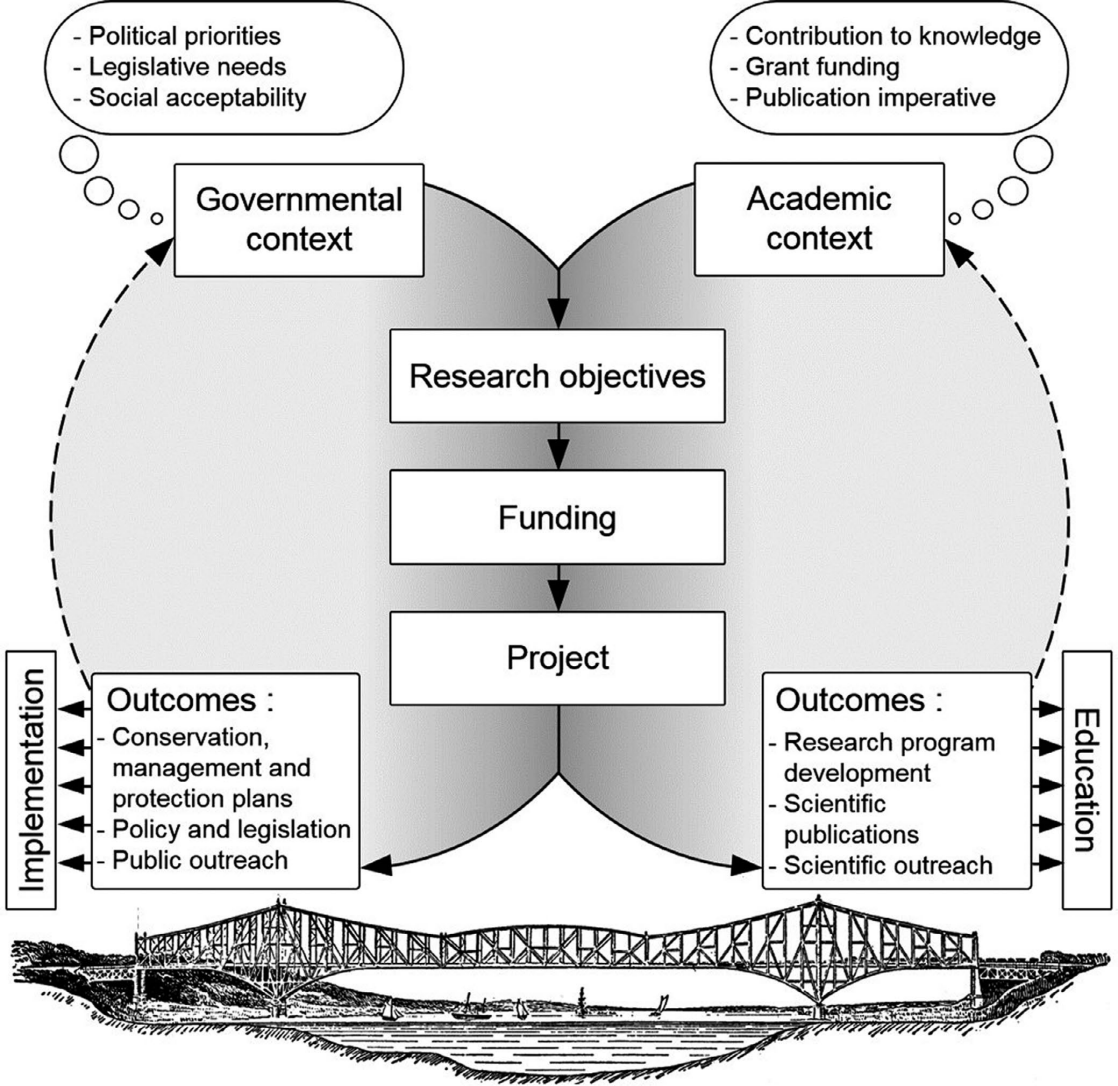
(Box 2). Schematic representation of governing forces (thought bubbles), active collaboration steps (solid lines) and retroaction loop (broken lines) of applied research projects involving government and academia, bridged by active communication and collaboration

Applying the concepts of evolutionary biology to wildlife management, conservation and forensics requires a well‐informed knowledge base of concepts and theory. Only then may practitioners select the most appropriate method to inform a decision‐making process and understand a method's limitations. Numerous evolutionary biologists having such expertise work within our department, many of whom trained in Dr. Louis Bernatchez's laboratory; these highly trained biologists now embody a sturdy bridge between academic research and policy‐driven implementation (Box 2, Figure 2). Throughout his brilliant career, Louis Bernatchez has acted as a pioneer, then became a prolific collaborator and now continues to thrive as a driver of innovation for the wildlife department of Quebec's provincial government. Along the way, he helped bridge the research–implementation gap through diverse and concrete applications of evolutionary biology concepts to local wildlife issues. The following sections present our department's focus on wildlife management, conservation and forensics by putting forward key evolutionary biology concepts and concrete examples and outcomes of their successful implementation that derive directly or indirectly from Louis Bernatchez's collaboration with our department (Figure 1).

Box 2 **Research and innovation in a governmental context**

One of the aims of this paper is to offer a glimpse of the current contributions of evolutionary applications in fulfilling wildlife management goals within our department. Evolutionary applications, along with other innovative science fields (i.e., genomics, biogeochemistry and telemetry), provide tangible decisions and are integrated increasingly into our frameworks. Generally, such theoretical considerations and technologies are integrated into these frameworks after a developmental phase (in collaboration or not) provided by fundamental research. Several factors can make the contribution of these innovations “silent” after being taken up into wildlife management, leading to an apparent disconnect between fundamental and applied practitioners. The forces governing success in academic and governmental contexts act differently in terms of their focus and scale (Figure 2). The feedback loop of needed publications and grant funding (i.e., “publish‐or‐perish”) is largely reduced in a governmental context, but it is replaced by a heightened importance for the agencies’ responsibilities and imperatives, legal obligations, political decisions and social acceptability. Hence, contingencies and annual schedules (e.g., annual surveys, policymaking or advisory committees) may hinder the contribution of wildlife management agencies within academic research projects. As already expressed in the “gap in application of genomics in conservation” debate (Garner et al., 2016; Shafer et al., 2015), a potential facilitator for integrating cutting‐edge technology in conservation and management can pass by active collaboration between government and academia, as exemplified here by the Bernatchez laboratory and MFFP partnership.

## 2 EVOLUTIONARY APPLICATIONS IN THE MANAGEMENT OF EXPLOITED WILDLIFE SPECIES

In our specific context, the contribution of evolutionary biology to the management of exploited wildlife species falls into two broad categories. The first is the greater recognition of issues directly linked to the evolution of exploited species; this includes, although not limited to, cases of reduced genetic diversity (e.g., Valiquette, Perrier, Thibault, & Bernatchez, 2014) and artificial selection (e.g.,, Conover & Munch, 2002). The second is the development of new genetic tools that have challenged more traditional approaches (e.g., tagging studies) and have pushed the boundaries of knowledge related to wildlife management; this includes using approaches to understand animal movement (e.g., Beacham et al., 2019), the relationship between parents and offspring (e.g., Richard, Dionne, Wang, & Bernatchez, 2013) and estimates of population abundance (Ferchaud et al., 2016; Roy, Albert, Bernatchez, & MRNF., 2007). For our department, these two categories of contributions have guided the implementation of fishing rules and stocking programmes on numerous occasions.

### 2.1 Harvest management

Population genetics and genomics have proven to be powerful tools for assessing a population's structure, a fundamental aspect for optimizing management measures. Such analyses can be used to identify and map appropriate management units. This information is crucial to implement adapted exploitation and conservation plans, as the geographic distribution of populations and their level of connectivity are tightly linked to stock abundance and extinction risk (O'Grady, Reed, Brook, & Frankham, 2004; Purvis, Gittleman, Cowlishaw, & Mace, 2000). In Quebec, analyses of population structure have been undertaken for the majority of the most socio‐economically important freshwater fishes.

#### 2.1.1 Population structure for an improved fishery management

Management of the recreational angling of landlocked Atlantic salmon (*Salmo salar*, Linnaeus 1758) in Lake Saint‐Jean (Quebec) clearly benefited from population structure studies. Tessier, Bernatchez, and Wright (1997) demonstrated that landlocked salmon spawn in four different tributaries, each formed genetically distinct populations with a sympatric feeding phase in the lake. Using fish caught by anglers in the various sectors of the lake, Potvin and Bernatchez (2001) showed a nonrandom spatial distribution of populations that generally remained stable over time. A dynamic and flexible fishery management framework emerged from that information; for instance, if one population is declining while the others are stable or increasing, managers can protect the vulnerable population by implementing fishery restrictions in tributaries and areas of the lake where this population mainly resides and allows anglers to keep practising their activity in other tributaries and sectors of the lake.

#### 2.1.2 Consequences of harvesting and management rules

The concept of harvest‐induced evolution is important for adequately managing fishery and hunting activities (Conover & Munch, 2002). For instance, fisheries targeting large individuals may induce artificial selection for early maturation at smaller sizes, leading to reduced fecundity and fisheries yield (e.g., Young et al., this issue). For Atlantic salmon, the mandatory release of all large size individuals that have been applied for many years in most exploited Canadian rivers may also lead to artificial selection considering that age and size at maturity have a genetic basis (Ayllon et al., 2015; Barson et al., 2015; Kusche et al., 2017). Therefore, to reduce the risk of fishery‐induced evolution, a river‐by‐river management approach applied in Quebec (MFFP, 2016b) stipulates that for healthy populations, both small and large salmon can be moderately harvested.

#### 2.1.3 Use of genetic information in estimates of population abundance

Analyses of population abundance have also benefited from genetics (Taberlet et al., 1997). For example, obtaining robust density estimates using conventional survey techniques, including classic mark–recapture studies, have been shown to be challenging for American black bear (*Ursus americanus,* Palas 1780). In this context, the noninvasive genetic sampling of hair collected in the field has heightened our knowledge of American black bear abundance by using an adapted mark–recapture approach based on individual identification (Dussault, Massé, Dumont, Lefort, & Cameron Trudel, 2016; Plante, Dussault, Massé, & Lefort, 2014; Roy et al., 2007). Movements of American black bears have also been studied using other population genetic statistics, such as Mantel tests and spatial autocorrelation analyses, to reveal a negative density‐dependent dispersal pattern (Roy, Yannic, Cote, & Bernatchez, 2012). Such genetic‐based mark–recapture approaches have now been used in different regions to monitor this species and guide management measures.

### 2.2 Stocking programmes

The improvement of stocking practices has relied greatly on numerous studies that suggested stocking could affect the integrity and diversity of populations (e.g., Marie, Bernatchez, & Garant, 2012; Morissette, Sirois, Wilson, Laporte, & Bernatchez, 2019; Perrier, April, Cote, Bernatchez, & Dionne, 2016; Tessier et al., 1997). Some of these studies helped to establish the relationships between population genetic parameters and various external factors (Gossieaux, Bernatchez, Siroi, & Garant, 2019; Marie et al., 2012; Valiquette et al., 2014). Other studies evaluated the effect of stocking on gene expression (Lamaze, Garant, & Bernatchez, 2013), intraspecific trophic niche partitioning (Morissette, Sirois, Lester, Wilson, & Bernatchez, 2018; Morissette et al., 2019), epigenetic programming (Le Luyer et al., 2017) and demographic gain (Milot, Perrier, Papillon, Dodson, & Bernatchez, 2013). The information acquired from these studies has been used to establish the stocking standards and guidelines of our department.

#### 2.2.1 Improving stocking practices

In an effort to assess the potential genetic alterations associated with Quebec stocking practices, Valiquette et al. (2014) demonstrated that levels of admixture in lake trout (*Salvelinus namaycush*, Walbaum 1792) populations were strongly correlated with stocking intensity; they identified a threshold value where genetic homogenization between the source and stocked populations could be expected. On the other hand, they also suggested that the genetic consequences of stocking could be temporary, as populations could experiment a genetic “purge” of exogenous alleles if no more stockings are conducted. Consequently, the new MFFP guidelines for lake trout states that populations should only be supplemented using a local source if they have been subjected to fewer than 15 stocking events and none of these events had a density >74 individuals/ha, as these populations are likely to have conserved their genetic integrity and must be protected. For lake trout populations having been subjected to more than 15 stocking events using an exogenous source or lakes that have experienced intense stocking events (≥74 individuals/ha), supplementation with fish from another lake may be allowed in some instances to enhance angling success, given that such heavily stocked populations have already lost their genetic integrity and may attract anglers that would have otherwise targeted wilder populations.

#### 2.2.2 Monitoring stocking efficiency

Molecular data can be used to assess the reproductive fate of stocked individuals and therefore the longer term demographic effect of enhancement activities. While numerous studies using tagging approaches have shown that stocked Atlantic salmon have a reduced survival rate (e.g., Jonsson, Jonsson, & Hansen, 2003), the use of genetics and heritability theory allowed a comparison of the reproductive success of stocked and wild fish. Milot et al. (2012) used molecular parentage analysis to assess the reproductive success of wild‐ and hatchery‐born Atlantic salmon. They genotyped all hatchery breeders, returning adults, and fry over consecutive cohorts. The relative reproductive success of fish born in hatcheries was nearly half that of wild‐born fish. Relative reproductive success varied with life stage, being 0.71 for fish released at the fry stage and 0.42 for fish released as smolt. These results presented a comprehensive assessment of the impact of stocking and supported the adoption of more natural rearing conditions for captive juveniles and their release at a younger stage (e.g. fry).

## 3 EVOLUTIONARY APPLICATIONS IN WILDLIFE CONSERVATION

Among the most recent and meaningful contributions of evolutionary biology to conservation, there has been growing consideration of biological units in need of conservation status beyond that of species units (Nielsen, 1995). Thus, our conservation objectives have expanded to include such vision. More recently, the exponential growth of mitochondrial genome databases has increased our ability to identify and detect cryptic and rare species as well as marked intraspecific diversity (de Santana et al., 2019; Kekkonen & Hebert, 2014). A critical assessment of the evolutionary processes that shape biodiversity has led to smarter recovery efforts, providing the tools to conserve not only the relevant biological units but also their evolutionary capacity (Hoffmann et al., 2015). These modern recovery efforts have occurred, most notably by use of genetically coherent supplementation programmes (Scheuerell et al., 2015; Williams & Hoffman, 2009).

### 3.1 Biodiversity conservation unit

The guidelines of the Convention on Biological Diversity state that biodiversity is recognized at three levels: the ecosystem, species and genetic levels. The recognition of these multiple levels implies a strong recognition of the intrinsic values of units along the species to populations (e.g., intraspecific) continuum (Coates, Byrne, & Moritz, 2018). Hence, the Canadian Species at Risk Act (SARA) can consider status assessments for below‐species conservation units, the *designatable unit*, to any “subspecies, varieties or geographically or genetically distinct population” shown to be critical to the evolutionary legacy of a biological species, and deemed irreplaceable through natural dispersal. Knowledge of evolutionary history has also greatly affected our understanding of speciation processes and their contemporary consequences (Garant, Forde, & Hendry, 2007), but also our understanding of how to preserve biodiversity. This is especially true for fish diversity where potential gaps in species designation and plausible areas of independently evolving lineages have been identified (April et al., 2011).

#### 3.1.1 Conservation of rainbow smelt populations in the St. Lawrence estuary

The consequences of postglacial colonization events have greatly shaped the genetic landscape in northern temperate fish species (Hocutt & Wiley, 1987; Mandrak & Crossman, 1992), including the existence of units of interest for conservation that would be ignored without proper genetic assessment, namely because of the lack of striking phenotypic and/or ecological variations. Analysis of mitochondrial DNA demonstrated that the St. Lawrence estuary represents a zone of secondary contact between two rainbow smelt (*Osmerus mordax*, Mitchill 1814) glacial races (Bernatchez, 1997). Genetic assessments show the existence of four distinct anadromous rainbow smelt populations (Bernatchez & Martin, 1996) that have, despite high spatiotemporal overlap, a very limited gene flow between the north and south shore populations (Baby, Bernatchez, & Dodson, 1991; Bernatchez, 1997; Pigeon, Dodson, & Bernatchez, 1998). The genetic uniqueness of the estuary's south shore populations, along with the increasing anthropogenic pressure and this population's steady decline (Giroux, 1997) has made the advisory committee designate these populations as “vulnerable” according to the Quebec Act respecting threatened or vulnerable species, a status provided in 2005. The re‐establishment committee has conducted significant conservation and restoration activities, formalized through the restoration action plan (Équipe de rétablissement de l'éperlan‐arc‐en‐ciel, 2003, 2009). Actions include habitat protection and restoration, the study of the species biology, a supplementation programme and citizen awareness. Recent work suggests that the population decline has halted, and signs of population recovery have even been observed (G. Verreault, personal communication). Without proper recognition of the unique character of these populations, it is quite likely that the St. Lawrence estuary south shore rainbow smelt populations, and their unique evolutive lineage, would have gone extinct.

#### 3.1.2 Considering the genetic structure among ecotypes in species management

Intraspecific evolutionary divergence (e.g., ecotypes or ecomorphs) is increasingly considered within management frameworks. Over the last two decades, genetic and genomic inputs have served as intricate proxies to study or integrate the relationships acting in caribou (*Rangifer tarandus,* Gmelin 1788) at the ecotype, herd and subpopulation levels for management and conservation strategies. Courtois, Bernatchez, Ouellet, and Breton (2003) demonstrated that the three caribou ecotypes present in Quebec (e.g., mountain, boreal and migratory) form distinct genetic entities. The authors also suggested that the different boreal caribou populations may form a metapopulation. They therefore formulated conservation recommendations based on the ecotypes’ genetic structure. Further investigation of ecotype connectivity showed an asymmetrical migration from migratory to boreal populations (Boulet, Couturier, Côté, Otto, & Bernatchez, 2007). This finding lead Yannic et al. (2016) to identify potential management units in eastern Canada (mostly Quebec and Labrador) by combining ecology and genetic structure. These studies, which focused on ecotype and population connectivity, have contributed significant amounts of knowledge to our department leading to a better understanding of intraspecific ecology and adapted management actions. At present, the shift to population‐specific parameters offers important insights into conservation targets (Gagnon, Yannic, Perrier, & Cote, 2019), a trend that is also considered for other species, such as for lake trout (Morissette et al., 2018) and brook trout (Crespel, Bernatchez, Audet, & Garant, 2013).

### 3.2 Recovery of threatened species

Whereas supplementation and supportive breeding are beneficial tools for preventing local extinction and assisting with species recovery, potential negative genetic impacts remain probable and could have adverse short‐ and long‐term consequences (Scheuerell et al., 2015; Williams & Hoffman, 2009). Studies of supplementation via evolutionary biology and genetics highlight that among potential negative consequences, the loss of genetic diversity, increased inbreeding and a loss of local adaptations are the key issues to address (Neff, Garner, & Pitcher, 2011). Management of threatened species through supportive breeding should therefore aim to minimize these negative effects to maximize evolutionary potential and avoid any erosion of fitness attributed to long‐term supplementation (Araki, Berejikian, Ford, & Blouin, 2008).

#### 3.2.1 Conservation of the copper redhorse through evolutionary coherent supplementation

The conservation and recovery of the copper redhorse (*Moxostoma hubbsi*, Legendre 1952) in Quebec are due in many respects to contributions of conservation genetics. The copper redhorse is a catostomid fish species, endemic to the province of Quebec; its distribution range comprises the St. Lawrence River and some tributaries, including the Richelieu River where its only currently known and remaining spawning sites are located (Dumont, Leclerc, Allard, & Paradis, 1997; Mongeau, Dumont, & Cloutier, 1992). Given the combined effect of habitat fragmentation, low recruitment and low abundance, the species is considered “endangered” by both the SARA and the Committee on the Status of Endangered Wildlife in Canada (COSEWIC). Accordingly, a supportive breeding programme was established to increase copper redhorse abundance while maintaining genetic diversity (Bernatchez, 2004). A study of the genetic population structure of the copper redhorse highlighted that despite a reduced abundance and the absence of population structure (one single spawning stock), high genetic diversity was remaining and low inbreeding was observed (Lippé, Dumont, & Bernatchez, 2006). These observations are suggested to be linked to the species’ long generation time and a gradual population decline that provides better retention of genetic diversity than would a population bottleneck (Amos & Balmford, 2001; Kuo & Janzen, 2004). Under the support programme, three million larvae and 140,000 juveniles were released in the Richelieu River between 2004 and 2009. Given the long life cycle of the species, it is still too early to quantify the effect of the supplementation programme; however, the young‐of‐the‐year recapture rate and the abundance of spawning individuals contributing to the supportive breeding programme show positive results (N. Vachon, personal communication).

## 4 EVOLUTIONARY APPLICATIONS IN WILDLIFE FORENSICS

The previous sections of this paper have presented concrete examples where evolutionary applications play a crucial role in defining recommendations for the sustainable exploitation of natural resources and biodiversity conservation. When recommendations are embedded into law and their pertaining regulations, law enforcement comes into play and closes the wildlife management loop by ensuring compliance. The use of molecular applications, as key tools in investigations or prosecutions of crimes involving wildlife, has spread worldwide over the last decades (Alacs, Georges, FitzSimmons, & Robertson, 2010; UNODC, 2016). Wildlife officer's interrogations addressed to forensic geneticists almost always deal with species identification, individual identification, parentage testing and population assignment. Evidence presented in forensic reports help officers at all stages of their investigations and result in higher law enforcement efficiency. In Quebec, the MFFP has been active in wildlife forensic science since the 1970s. Genetic analyses were introduced into the routine workflow in the last decade. This innovative development was consequent to a fruitful partnership with Dr. Louis Bernatchez and widened the laboratory's services essentially by enabling species identification for a much broader spectrum of species, surging the statistical power of individual identification analysis and adding sex identification. The following sections discuss each type of analysis and present some of the related work conducted within our department.

### 4.1 Forensic species identification

Since wildlife legislations are usually specific to species or groups of species, in most investigations, it is essential for wildlife officers to identify the originating species of biological evidence. When identification based on morphological characteristics is not possible, wildlife forensic practitioners usually turn to Sanger sequencing of specific mitochondrial DNA (mtDNA) regions (Linacre & Tobe, 2009). No consensus exists in the wildlife forensic community with different laboratories and even different taxonomic groups within a laboratory relying on different mtDNA regions.

#### 4.1.1 Identification of mammals, fishes and birds using DNA barcoding

The MFFP’s provincial wildlife forensic laboratory has been processing evidence for forensic species identification since the very beginning, mostly relying on immunochemical and biochemical analyses at first (Mardini, 1984). In 2009, Sanger sequencing was implemented and a cytochrome *c* oxidase I region, also referred to as the DNA barcode (Dawnay, Ogden, McEwing, Carvalho, & Thorpe, 2007), was identified as the region of interest. Extensive databases for mammal, fish and bird species have been assembled mostly from publicly available data (e.g., April et al., 2011) which significantly widened the range of identifiable species. Moreover, the robustness of the technique now enables species identification on most of the 500 evidences submitted yearly, from high‐quality DNA samples, such as meat and fish fillet, to low‐copy number or low‐quality samples, such as hair, blood and other trace evidence. The most frequent species observed are species subjected to fishing and hunting in the province. Less common species are also encountered on a regular basis; these are mostly from native wildlife and domesticated species, including pets and farm animals. For instance, appropriate charges can be laid after counting and identifying the species of origin for a bundle of fish filet or illegal possession of protected species such as birds of prey can be proved using a single feather.

### 4.2 Forensic individual identification

Individual identification is crucial to many wildlife forensic cases that seek to answer if different exhibits come from the same animal. The development of wildlife individual profiling panels is a resource‐intensive task. First, panels are species‐specific and few consensus panels exist, as most are developed for specific research objectives, not necessarily having in mind the forensic context requirements and goals. Wildlife genomes are also less studied, making the informative marker discovery process laborious and limiting the statistical power of panels. Finally, representative samples for population studies in a forensic context are challenging to collect, especially for rare species or species with distribution range located in remote areas. Combining these factors with global investments in wildlife forensics receiving less attention than human‐related forensic disciplines helps explain why laboratories continue to work independently, mostly with unpublished panels and data sets validated for only a few of the most common species. Nonetheless, success stories in individual identification within wildlife forensics can be found in the literature (e.g., Jobin, Patterson, & Zhang, 2008; Lorenzini, 2005), and some thorough validation studies have been published (e.g., Ciavaglia & Linacre, 2018; Dawnay et al., 2008; van Hoppe, Dy, Einden, & Iyengar, 2016).

#### 4.2.1 Individual identification tools

In the past, our department's wildlife forensic laboratory has performed individual identification of moose (*Alces alces*, Linnaeus 1758) and white‐tailed deer (*Odocoileus virginianus*, Zimmermann 1780) using allozymes and having an extremely limited statistical power. In 2007, microsatellite panels and provincial population genetic databases were developed (Albert, Côté, & Bernatchez, 2007). The microsatellite variability within moose and white‐tailed deer populations increased the statistical power of individual identification analysis markedly compared to that of the previously used allozymes. Following this improvement, wildlife officers’ requests evolved from mostly species identification to a combination of species and individual identification. Since then, microsatellite panels have been developed for American black bears and caribou, thus covering our jurisdiction's big‐game species. This application is requested for more than 250 pieces of evidence per year. Its value is well recognized, mostly in poaching and trafficking casefiles requiring a demonstration that different pieces of evidence all come from the same animal, such as viscera found at an illegal killing site, blood collected on a vehicle and meat seized in a freezer.

The MFFP also uses individual identification via molecular markers for investigations where the perpetrator is an animal. When a wildlife attack on a human is reported, such as a coyote (*Canis latrans*, Say 1823) or American black bear attack, wildlife officers submit, when available, biological evidence collected at the site of an attack (e.g., the victim's clothes with bite marks, hairs and faeces) to compare the evidence with samples collected from a suspected aggressor captured afterwards. Finding an individual match allows the search for the aggressor to be halted and provides a renewed sense of safety for the neighbouring community.

### 4.3 Forensic population identification

In many wildlife forensic cases, due to regional disparities in regulations, species identification is not sufficient and the geographic origin of a specimen is required. The development of tools for identifying the geographic origin of a specimen in a forensic context remains in its early stages for most species (Ogden & Linacre, 2015). Hence, standards and guidelines are lacking for this particular application. Nonetheless, the great potential of this application was demonstrated in multiple African elephant (*Loxodonta africana*, Blumenbach 1797) ivory cases (Ishida, Georgiadis, Hondo, & Roca, 2013; Wasser et al., 2015). Depending on the required resolution and the genetic divergence between the groups, different types of molecular markers are considered (Alacs et al., 2010; Ogden, 2008, 2009). When comparing populations or regional groups that show important genetic differences, direct observation of fixed haplotypes or genotypes associated with the geographic regions of interest could be sufficient (Sanders et al., 2008; Summerell, Frankham, Gunn, & Johnson, 2019). When fixed haplotypes or genotypes are not observed, the identification of a geographic origin then relies on differences in allele frequencies between populations in nuclear DNA markers, such as microsatellites or SNPs (Horreo, Machado‐Schiaffino, & Garcia‐Vazquez, 2017; Karmacharya et al., 2018; Mondol, Sridhar, Yadav, Gubbi, & Ramakrishnan, 2015; Nielsen et al., 2012; Pukk, Gross, Vetemaa, & Vasemagi, 2016; Schwenke, Rhydderch, Ford, Marshall, & Park, 2006).

#### 4.3.1 Ecotype identification of caribou

In Quebec, specific designations and their pertaining regulations apply to the boreal and mountain (Gaspésie population only) ecotypes of caribou (Section 3.1.2). From a province‐wide database of more than 500 caribou genotyped for 16 microsatellite markers (Yannic et al., 2016), the power of population assignment analysis was evaluated for the genetic distinction of ecotypes. The analysis revealed that microsatellite markers reassigned caribou samples to their correct ecotype at a rate of >95%; however, confidence in these assignments was often low, thus not providing strong enough evidence for a court setting. In our aim to develop a more powerful population assignment tool, these results led our department to evaluate other, more sensitive applications. To this end, an ongoing project in collaboration with a research team at Laval University aims to develop a SNP chip and web portal for relatively easy sample processing and result interpretation (caribougenomics.org). Once the validation study is completed, this new tool should serve in investigations where identification of the caribou ecotype is needed.

## 5 PERSPECTIVES FOR FURTHER AND FUTURE IMPLEMENTATIONS

Building on these successes, the MFFP is moving forward in implementing more evolutionary coherent practices for wildlife conservation and management. Considering evolutionary coherent management practices in our future management plans means to minimize the negative impacts consequent to harvest regulations and stocking programmes. To this end, harvest size range is now scrutinized to limit human‐induced evolution and maintain intact evolutionary trajectories. In addition to limit artificial selection, we now have strong support to encourage a proper angling experience which could satisfy our most enthusiastic sports fishermen and preserve vulnerable fish populations. Before Richard et al. (2013), few studies had assessed the reproductive success (and fitness) of fish subject to catch and release. Such information is nevertheless crucial, considering that each year over 100,000 Atlantic salmon are caught worldwide and, for conservation purposes, are released (ICES, 2019). They concluded that the mean reproductive success of caught and released salmon did not differ significantly from uncaptured salmon, confirming to managers and anglers that catch and release can be a very effective management tool for Atlantic salmon.

Integrating evolutionary concepts into management strategies does not always necessitate conducting our own long‐term monitoring studies, but could gain from scientific developments to improve management practices. For example, we used the model developed by Ryman and Laikre (1991) to refine our stocking guidelines for Atlantic salmon in order to deal with the inevitable compromise between demographic gain and effective population size loss. Moreover, theory predicts that effective population size over a generation (Ne) and effective population size over a reproductive cycle (Nb) can inform us about the rate of decline of genetic diversity and can be traced back in time. Ferchaud et al. (2016) investigated the relationships between Ne, Nb and census size (Nc) in 10 Atlantic salmon populations over time. Results confirmed a positive correlation between the three parameters, suggesting Nb as a potentially reliable parameter for tracking Ne and Nc. Knowledge obtained from these evolutionary concepts has been used to improve rearing practices and establish new conservation limits directly guiding Atlantic salmon fishing management rules (MFFP, 2016b). Based on this knowledge, we could also adjust our future surveys to collect noninvasive samples and use less labour‐intensive methods to estimate population abundance and genetic diversity.

From these established evolutionary principles, our department is able to transition into a more comprehensive application of evolutionary biology and broaden the scope of application to new ramifications among contemporary conservation issues. Over the last decades, increased international transits and climate change have facilitated the spread and dispersion of novel parasites and pathogens (Hellmann, Byers, Bierwagen, & Dukes, 2008; Tompkins, Carver, Jones, Krkosek, & Skerratt, 2015; Van Hemert, Pearce, & Handel, 2014). Given the socio‐economic importance of wildlife and livestock agriculture, the ecological value of biodiversity and human health issues, management interventions are needed in disease and biosecurity management. Integrating evolutionary principles and appropriate applications are critical for maximizing the success of disease prevention, surveillance, control and eradication. Our department contributes to the Canadian Wildlife Health Cooperative, a nationwide network dedicated to wildlife health. This network monitors emerging or prevalent diseases and takes appropriate management actions (e.g., oral rabies vaccination of wildlife to prevent rabies outbreak and white‐tailed deer culling to control and prevent chronic wasting disease spread; MFFP, 2016a). Another example is the application of evolutionary principles, such as host landscape genetics, when designing the best management strategies to predict the potential dispersal route of variants of raccoon rabies (Paquette, Talbot, Garant, Mainguy, & Pelletier, 2014) or the whole‐genome phylogeography of pathogens to understand the underlying processes of an outbreak (Trewby, Nadin‐Davis, Real, & Biek, 2017). The recent integration of evolutionary principles in wildlife disease management should lead the way towards a further contribution of evolutionary applications in this field.

**FIGURE 3:**
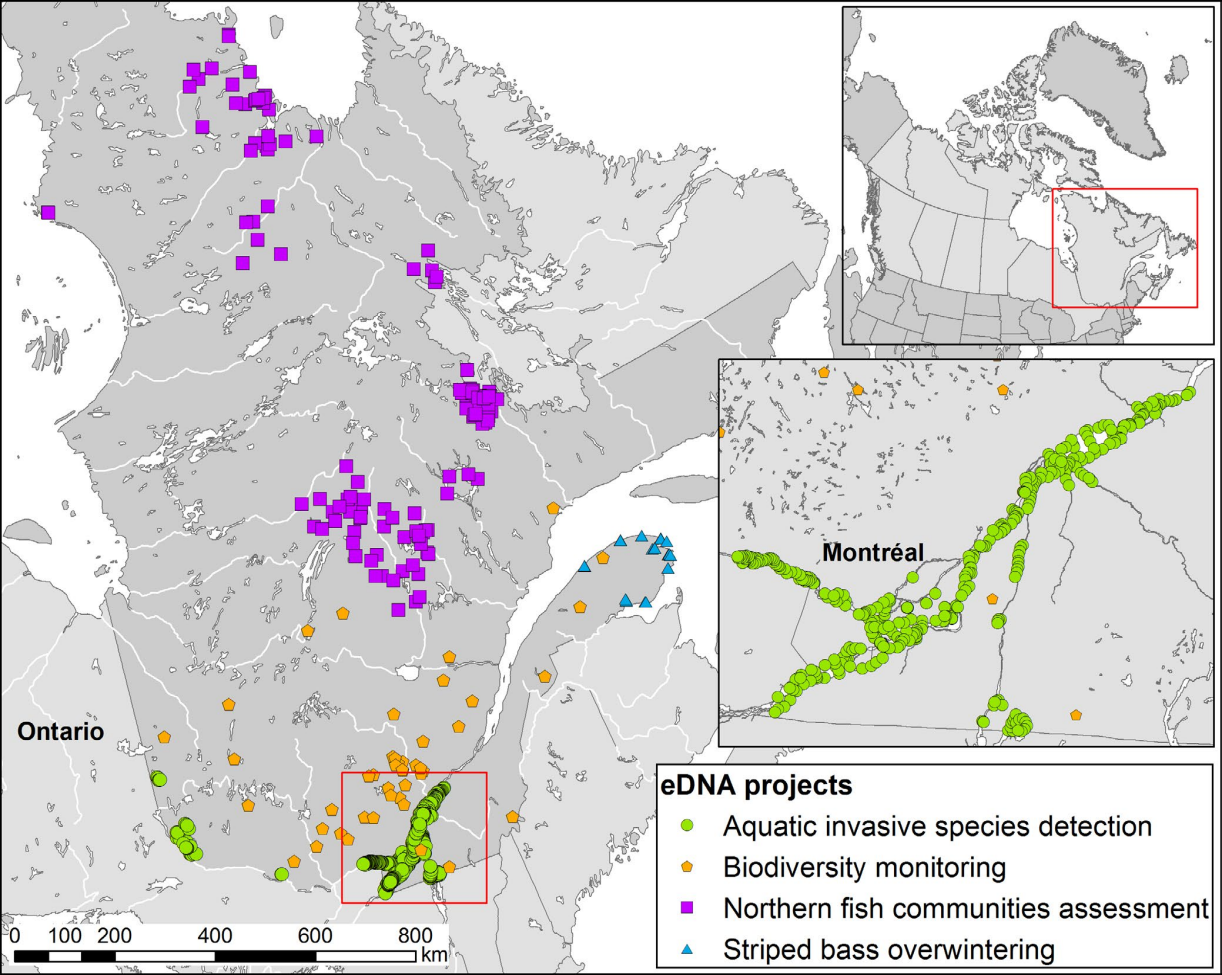
Province‐wide use of eDNA for the detection of aquatic invasive species (green circle), biodiversity monitoring (orange pentagons), the assessment of northern fish communities (purple squares) and surveys of the overwintering habitats of striped bass (*Morone saxatilis*, Walbaum 1792)

The fastest growing applications of molecular biology, and conveniently some of the latest remarkable contributions of Dr. Bernatchez's collaboration with our department, are methods derived from the detection and quantification of eDNA. Invasive species are identified as the second greatest threat to biodiversity after habitat destruction (Bellard, Cassey, & Blackburn Tim, 2016). Hence, management of invasive species is a growing concern and MFFP is responding by including it among its mandates (Figure 3). The early detection of invasive species allows managers to act quickly to control or eradicate invasive species, but also to identify and predict potential dispersal routes when complemented with other evolutionary concepts. Furthermore, as our department expands its mission to northern and remote regions (Figure 3), eDNA will continue to be a part of bioassessment protocols. Notwithstanding the already abundant use of eDNA, our department is aiming to refine methods of estimating the abundance of various species from lake water samples (Lacoursière‐Roussel, Rosabal, & Bernatchez, 2016). Although concerns exist for this application, owing to the challenges in quantifying and assessing the environmentally specific rates of eDNA decay, the implementation of such an application would be quite valuable (Bylemans, Furlan, Gleeson, Hardy, & Duncan, 2018; Lacoursière‐Roussel, Dubois, Normandeau, & Bernatchez, 2016; Lodge et al., 2012).

As we reflect on the past and present contributions of evolutionary biology to wildlife management, conservation and forensic applications, the obvious future perspective shared among these fields is the potential held by next‐generation sequencing (NGS) technologies and, more broadly, the use of ‐omic frameworks. Indeed, genomics has compelling possibilities with the increased resolution of traditional genetic metrics that could refine fisheries management, abundance estimation models, monitoring of stocking programmes, mixed‐stock analysis and forensics (e.g., microhaplotypes; McKinney, Seeb, & Seeb, 2017; Oldoni, Kidd, & Podini, 2019). The promises of genomics to address adaptive divergence, functional gene–environment associations and wild population deleterious mutation loads seem to be fulfilled in some instances (Ferchaud, Laporte, Perrier, & Bernatchez, 2018). Whereas evidence of causal relationships with population dynamics or harvest is limited, likely preventing its widespread implementation in wildlife management and conservation (Bourret, Dionne, Kent, Lien, & Bernatchez, 2013; Shafer et al., 2015), its ability to decipher between neutral and adaptive divergence is key. This potential is likely to set new and optimized conservation priorities. Moreover, recent progress offers a promising future for NGS technologies in forensics as well (Borsting & Morling, 2015; de Knijff, 2019; Ogden, 2011). With the growing availability of wildlife and nonmodel species genomes, powerful wildlife forensic panels could become more accessible. Shorter fragments could be targeted to increase the success rate, as low‐template DNA samples are often encountered in forensics.

## 6 CONCLUSION

Overall, evolutionary applications in wildlife sciences have grown rapidly, and as demonstrated by examples of past and present collaborative effort between MFFP and academics, mainly Dr. Bernatchez, the implementation of evolutionary concepts had a major impact on critical conservation issues. Looking ahead, before genomic applications are widely used in wildlife conservation applications, important aspects remain to be addressed. For the wildlife forensic community, this revolves predominantly around the production of official standards and guidelines, as well as establishing criteria for all possible technological, interpretation and reporting issues. We argue that just as what we considered novel genetic methods 20 years ago and now consider “traditional” and “standard,” present‐day genomic approaches will quickly prove their potency in solving challenging diverse conservation issues, democratizing such technology again. From our standpoint, it is clear that as long as practitioners continue to collaborate and maintain awareness of academic research by asking the right questions, requesting the development of specific tools and adapting to genomic methods, genomic conceptual developments and applications will cross the bridge and make the leap into widespread implementation. The MFFP is very fortunate to benefit from this tight collaboration with academic researchers. Such a bridge was not built in a single day or one successful cooperation. Our advice to build a long‐term flourishing collaboration is for both parties to communicate their needs, act in transparency and seize all opportunities to develop projects together for the benefit of wildlife, science and future generations.

## CONFLICT OF INTEREST

None declared.

## Data Availability

Data sharing is not applicable to this article as no new data were created or analysed in this study.
